# Evaluation of plasma exosomal microRNAs as circulating biomarkers for progression and metastasis of gastric cancer

**DOI:** 10.1002/ctm2.171

**Published:** 2020-10-11

**Authors:** Yuqi Chen, Huang Feng, Yanlin Wu, Ruoqin Wang, Zhaoji Li, Jiajia Chen, Wenying Yan, Weichang Chen

**Affiliations:** ^1^ Department of Gastroenterology The First Affiliated Hospital of Soochow University Suzhou China; ^2^ School of Chemistry Biology and Materials Engineering Suzhou University of Science and Technology Suzhou China; ^3^ Center for Systems Biology Department of Bioinformatics School of Biology and Basic Medical Sciences Soochow University Suzhou China; ^4^ Jiangsu Key Laboratory of Clinical Immunology Soochow University Suzhou China; ^5^ Jiangsu Key Laboratory of Gastrointestinal Tumor Immunology The First Affiliated Hospital of Soochow University Suzhou China

Dear Editor,

Till now, abundant studies have shown that exosomal miRNA may have potential clinical application value as biomarkers for diagnosis and evaluation in gastric cancer (GC).^1^, ^2^ However, current researches not only rarely refer to plasma exosomal miRNAs (PEMs) for progression and metastasis prediction, but also seem to be limited to individual PEM identification, ignoring that miRNAs are often expressed in clusters to achieve regulatory functions. Thus inspiring us to conduct this systemic and comprehensive studies on PEMs to excavate their value in accurate evaluation of progression and metastasis in GC.

Plasma samples from 23 GC patients and six healthy controls (HC) were collected in this work, together with their clinical pathological features (Table S1). Our study workflow is shown in Figure S1. Exosomes from plasma samples were extracted by exoEasy Maxi Kit and identified by transmission electron microscopy (Figure 1A). After small RNA sequencing, PEMs were annotated and calculated (Figure 1B). We obtained 1853 PEMs, including 1114 known mature miRNAs and 739 predicted novel miRNAs. The principle component analysis (PCA) of all 1853 PEMs showed that the GC group and the HC group partially overlapped, and observable dispersion existed among the GC samples (Figure 1C). However, Figure 1D demonstrated that the above overlapping or dispersion could be eliminated and the GC group and HC group samples could be separated by the PCA of 31 differentially expressed PEM (dPEMs) selected by comparing the GC group and the HC group (Table S2).

**FIGURE 1:**
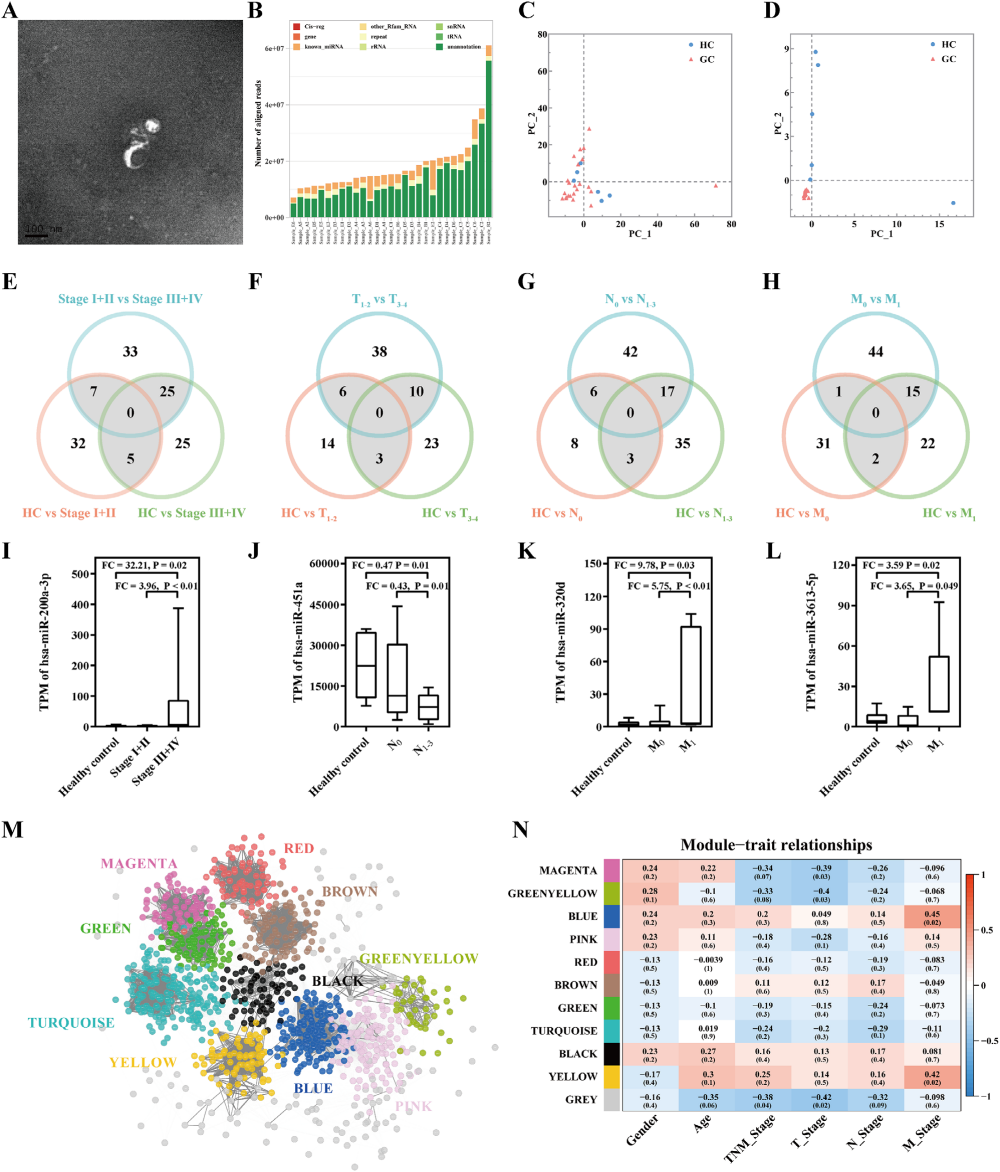
A, Transmission electron microscopy of exosomes. B, Annotation results of exosomal small RNAs. C, PCA including all identified 1853 PEMs. Samples from both the HC group and the GC group were dispersed and partly overlapped. D, PCA including 31 dPEMs in GC patients compared with healthy controls. Samples from the GC group concentrated and separated from the HC group. E‐H, Identification of dPEMs related to different clinical pathological features. Based on different clinical pathological features, pairwise comparison among different subgroups (TNM pathological staging: Stage I+II/Stage III+IV subgroups, primary tumor invasion depth: T_1‐2_/T_3‐4_ subgroups, lymph node metastasis: N_0_/N_1‐3_ subgroups, and distant metastasis: M_0_/M_1_ subgroups). Venn diagrams were performed to find dPEMs in the intersection area. I‐L, Transcripts per million (TPM) of four dysregulated PEMs related to clinical pathological features. M, Ten PEM co‐expressed modules were identified in the whole weighted network. Different modules are distinguished by different colors. Grey nodes do not belong to any module. N, Matrix of correlations among modules and clinical pathological features

To explore the relationship between PEMs and different clinical pathological features (including TNM pathological staging, primary tumor invasion depth, lymph node metastasis, and distant metastasis), PEMs were analyzed at both single PEM level and network level. Figure 1E‐H presented the numbers of dPEMs in each pairwise comparison following Venn diagram. All the known mature dPEMs that appeared in the intersection area of the Venn diagram are presented in Table S3. Comparing these dPEMs obtained in exosomes, it was found that nine dPEMs were also dysregulated in tumor tissues in the TCGA dataset (Table S4). Considering the ability to stably express among samples, a total of four dPEMs were finally selected (Figure 1I‐L). It is shown that exosomal hsa‐miR‐200a‐3p was upregulated in the Stage III+IV subgroup, and its expression was significantly higher than in the HC group and the Stage I+II subgroup. While exosomal hsa‐miR‐451a was downregulated in the N_1‐3_ subgroup, lower than in the HC group and the N_0_ subgroup. Considering distant metastasis, exosomal hsa‐miR‐320d and hsa‐miR‐3613‐5p were upregulated in M_1_ subgroup, higher than in the HC group and the M0 subgroup. Interestingly, these four dPEMs were all differentially expressed in the subgroups of more advanced stages of GC.

At the network level, a total of 10 PEM co‐expressed modules were identified by Weighted gene co‐expression analysis (Figure 1M; Table S5). Figure 1N reveals that modules BLUE and YELLOW were significantly positively correlated with distant metastasis (*Cor* = 0.45, 0.42, *P*‐value = .02, .02). As shown in Figure 2A,B, PEMs highly related to these two modules were consistently related to distant metastasis with *Cor* (MM in BLUE/YELLOW vs. GS for distant metastasis) = 0.60/0.64, *P*‐value (BLUE/YELLOW) = 2 × 10^‐17^ */*4 × 30^‐11^. Afterward, co‐expression networks of each module were built (Figure 2C,D), and hub miRNAs were identified (Table S6). All hub miRNAs in BLUE or YELLOW were significantly correlated with distant metastasis. Notably, hsa‐miR‐3613‐5p (hub miRNA in BLUE module) expression level in the distant metastasis samples were both higher than those in the HCs and GCs without distant metastasis. Thus, we selected hsa‐miR‐3613‐5p and its first order neighbors in the BLUE module as the core (Figure 2E), which contains other eight hub miRNAs and five members of miR‐371‐373 family (Table S7). Then, as shown in Figure 2F, the target genes of this core were highly enriched in PI3K‐Akt signaling pathway, MAPK signaling pathway, mTOR signaling pathway, Ras signaling pathway, FoxO signaling pathway, and TGF‐β signaling pathway, confirmed this core module's potential role in progression and metastasis of GC. Moreover, in GO annotation (Figure S2), the PEMs in the core were highly enriched in terms associated with the origin of exosome‐enclosed miRNAs. In YELLOW module, five miRNAs (Table S6) were identified as hub miRNAs and significantly correlated with distant metastasis, and several first order neighbors of them were significantly higher expressed in distant metastasis GC samples.

**FIGURE 2:**
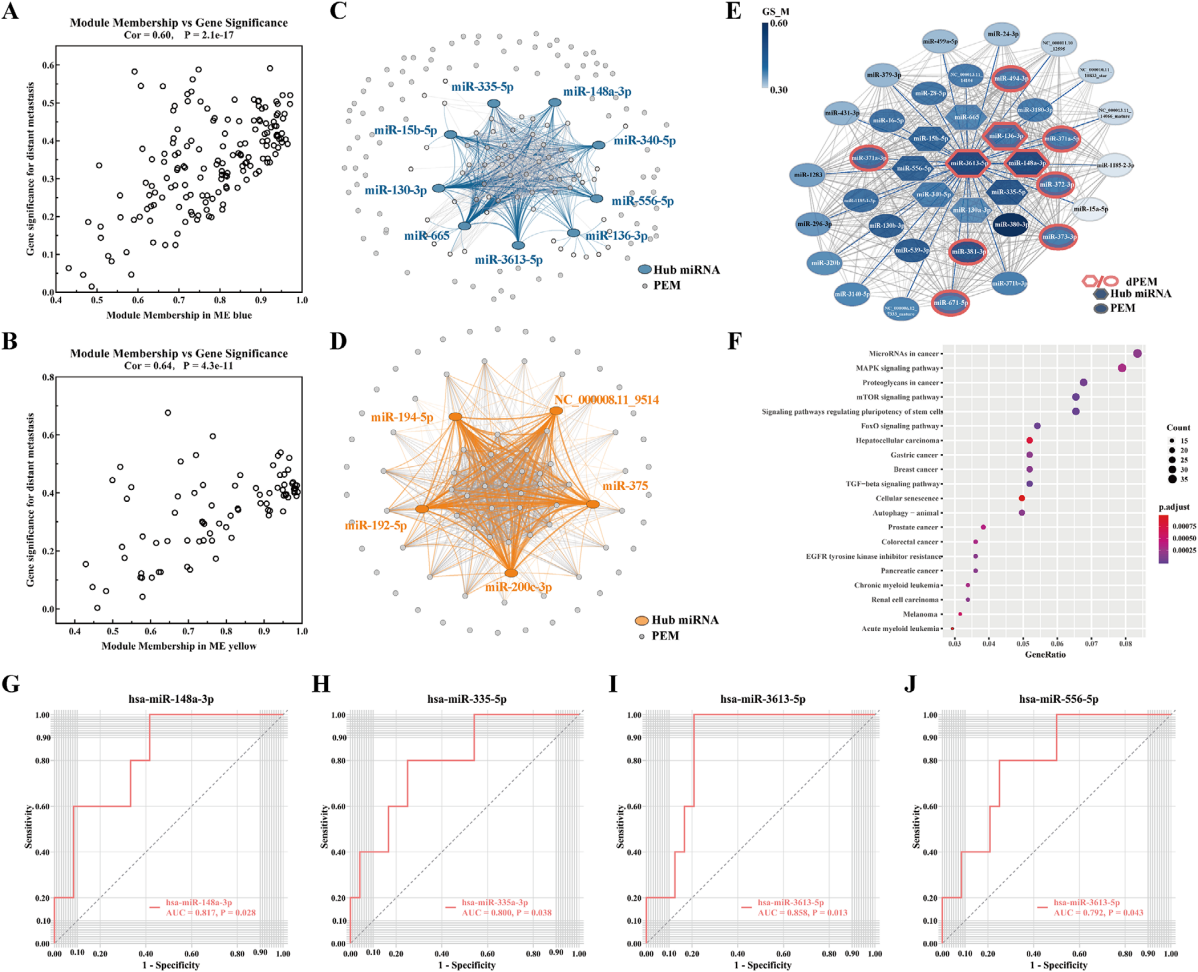
A,B, Correlation between MM and GS in BLUE and YELLOW. C,D, Co‐expression network in BLUE and YELLOW. E, The core contained hsa‐miR‐3613‐5p and its co‐expressed PEMs in BLUE. Each node in the figure represent a PEM. The node color was related to the GS associated with distant metastasis. F, Top 20 enriched KEGG pathway of target genes of exosomal hsa‐miR‐3613‐5p and its co‐expressed PEMs. G,J, ROC of hub miRNAs in BLUE module. ROC–AUC of hsa‐miR‐148a‐3p was 0.817 (0.638‐0.996, *P*‐value = .028). The optimal sensitivity and specificity of hsa‐miR‐148a‐3p was 100% and 58.3%, respectively, with a cut‐off value of 453.526. ROC–AUC of hsa‐miR‐335‐5p was 0.800 (0.601‐0.999, *P*‐value = .038). The optimal sensitivity and specificity of hsa‐miR‐335‐5p was 80.0% and 75.0%, respectively, with a cut‐off value of 159.144. ROC–AUC of miR‐3613‐5p was 0.858 (0.721‐0.995, *P*‐value = .013). The optimal sensitivity and specificity of miR‐3613‐5 was 100.0% and 79.2%, respectively, with a cut‐off value of 9.272. ROC–AUC of hsa‐miR‐556‐5p was 0.792 (0.602‐0.981, *P*‐value = .043). The optimal sensitivity and specificity of hsa‐miR‐556‐5p were 80.0% and 75.0%, respectively, with a cut‐off value of 5.256

Four hub miRNAs in BLUE module, including hsa‐miR‐148‐3p, hsa‐miR‐335‐5p, hsa‐miR‐3613‐5p, and hsa‐miR‐556‐5p, were identified as potential circulating biomarkers for distant metastasis in GC (Figure 2G‐J), with AUC = 0.817, 0.800, 0.858, and 0.792. Although we did not found modules closely related to pathological stage and lymph node metastasis, we still tried to explore the diagnostic value of exosomal hsa‐miR‐200a‐3p and hsa‐miR‐451a, considering that they were significantly dysregulated in the Stage III+IV subgroup and the N_1‐3_ subgroup, respectively (Figure S3).

Recent researches have shown that exosomes may be involved in the occurrence and development of several kinds of tumorous diseases and especially participate in the regulation of tumor microenvironments^3^, ^4^ and the establishment of a pre‐metastatic microenvironment.^5^ Thus providing that exosomes are more likely to participate in the progression and metastasis of GC, and have much more application value in early identification of these disease states related migration. Our work has discovered the potential mechanism and application value of exosomal has‐miR‐3613‐5p in GC, which was rarely reported. While for its highly co‐expressed miR‐371‐373 family, they have been reported to promote proliferation and progression of GC.^6^, ^7^ Furthermore, previous researches suggested potential application value of some non‐exosomal miRNAs in GC, such as miR‐200a^8^ and miR‐451a.^9^ Our work supplemented the expression characteristics and application prospects of these miRNAs in exosomes.

Conclusively, there existing some members of the PEMs that were closely related progression and metastasis in GC. And our data suggest that plasma exosomal hsa‐miR‐3613‐5p in particular appeared to be the most promising circulating exosomal miRNA biomarker for metastasis of GC, considering its potential value in biological actions and diagnosis of distant metastasis in GC.

## Supporting information

Supporting

Figure 1

Figure 2

Figure 3

## References

[1] Yang H , Fu H , Wang B , et al. Exosomal miR‐423‐5p targets SUFU to promote cancer growth and metastasis and serves as a novel marker for gastric cancer. Mol Carcinog. 2018;57(9):1223‐1236. 29749061 10.1002/mc.22838

[2] Ji R , Zhang X , Gu H , et al. miR‐374a‐5p: a new target for diagnosis and drug resistance therapy in gastric cancer. Mol Ther Nucleic Acids. 2019;18:320‐331. 31614322 10.1016/j.omtn.2019.07.025 PMC6796712

[3] Zhou W , Fong M Y , Min Y , et al. Cancer‐secreted miR‐105 destroys vascular endothelial barriers to promote metastasis. Cancer Cell. 2014;25(4):501‐515. 24735924 10.1016/j.ccr.2014.03.007 PMC4016197

[4] Luga V , Zhang L , Viloria‐Petit AM , et al. Exosomes mediate stromal mobilization of autocrine Wnt‐PCP signaling in breast cancer cell migration. Cell. 2012;151(7):1542‐1556. 23260141 10.1016/j.cell.2012.11.024

[5] Peinado H , Zhang H , Matei IR , et al. Pre‐metastatic niches: organ‐specific homes for metastases. Nat Rev Cancer. 2017;17(5):302‐317. 28303905 10.1038/nrc.2017.6

[6] Guo H , Ji F , Zhao X , et al. MicroRNA‐371a‐3p promotes progression of gastric cancer by targeting TOB1. Cancer Lett. 2019;443:179‐188. 30529155 10.1016/j.canlet.2018.11.021

[7] Zhang X , Li X , Tan Z , et al. MicroRNA‐373 is upregulated and targets TNFAIP1 in human gastric cancer, contributing to tumorigenesis. Oncol Lett. 2013;6(5):1427‐1434. 24179536 10.3892/ol.2013.1534 PMC3813807

[8] Zhou X , Wang Y , Shan B , et al. The downregulation of miR‐200c/141 promotes ZEB1/2 expression and gastric cancer progression. Med Oncol. 2015;32(1):428. 25502084 10.1007/s12032-014-0428-3

[9] Bandres E , Bitarte N , Arias F , et al. microRNA‐451 regulates macrophage migration inhibitory factor production and proliferation of gastrointestinal cancer cells. Clin Cancer Res. 2009;15(7):2281‐2290. 19318487 10.1158/1078-0432.CCR-08-1818

