# Supplementary material for: Evaluation of plasma exosomal microRNAs as circulating biomarkers for progression and metastasis of gastric cancer
Source: Clin Transl Med. 2020 Oct 11;10(6):e171. doi: 10.1002/ctm2.171 (PMC7548098; doi:10.1002/ctm2.171)
Supplement: Supplementary file 1 — Supporting [file CTM2-10-e171-s001.docx]

Table S1. Clinical pathological features of GC patients

| **Clinical Pathological Features** | **Numbers of Cases** |
| --- | --- |
| Pathological TNM stage |  |
| Stage I | 6 |
| Stage II | 6 |
| Stage III | 6 |
| Stage IV | 5 |
| ^*^ Primary tumor invasion depth |  |
| T_1-2_ | 9 |
| T_3-4_ | 10 |
| ^*^Lymph node metastasis |  |
| GC without lymph node metastasis ( N_0_ ) | 9 |
| GC with lymph node metastasis ( N_1-3_ ) | 10 |
| Distant metastasis |  |
| GC without distant metastasis ( M_0_ ) | 18 |
| GC with distant metastasis ( M_1_ ) | 5 |

^*^4 GC patients without surgical resection did not obtain gross histological specimens, so the primary tumor invasion depth and lymph node metastasis were not classified according to the pathological stage.

Table S2. 31dPEMs in GC patients compared with healthy controls

| **dPEM** | **FC** | **P value** | **Dysregulation** |
| --- | --- | --- | --- |
| hsa-miR-1249-3p | 0.111 | 1.73E-02 | Down |
| hsa-miR-1278 | 0.022 | 3.99E-02 | Down |
| hsa-miR-138-5p | 0.008 | 3.79E-02 | Down |
| hsa-miR-19b-1-5p | 0.000 | 4.53E-02 | Down |
| hsa-miR-27a-5p | Inf | 4.91E-02 | Up |
| hsa-miR-3174 | 0.102 | 3.01E-02 | Down |
| hsa-miR-33a-3p | 0.004 | 1.46E-02 | Down |
| hsa-miR-3661 | 0.000 | 7.31E-03 | Down |
| hsa-miR-3691-3p | 0.000 | 3.36E-02 | Down |
| hsa-miR-374c-5p | 0.010 | 4.87E-02 | Down |
| hsa-miR-3911 | 0.000 | 3.42E-02 | Down |
| hsa-miR-4474-3p | 0.000 | 2.06E-02 | Down |
| hsa-miR-4507 | 0.002 | 3.37E-02 | Down |
| hsa-miR-4520-3p | 0.000 | 3.70E-02 | Down |
| hsa-miR-6716-3p | 0.000 | 5.28E-03 | Down |
| hsa-miR-6772-5p | 0.000 | 4.49E-02 | Down |
| hsa-miR-7843-5p | 0.019 | 2.49E-02 | Down |
| NC_000001.11_1017 | 0.091 | 4.24E-02 | Down |
| NC_000002.12_2646 | 0.076 | 8.36E-03 | Down |
| NC_000004.12_5200 | 0.006 | 4.29E-02 | Down |
| NC_000004.12_5412 | 0.004 | 3.00E-02 | Down |
| NC_000007.14_8428 | 37.700 | 8.35E-03 | Up |
| NC_000008.11_9345 | 0.034 | 4.20E-02 | Down |
| NC_000009.12_9873 | 0.002 | 2.75E-02 | Down |
| NC_000009.12_10454 | 0.011 | 1.72E-02 | Down |
| NC_000014.9_14630_mature | 0.007 | 2.36E-02 | Down |
| NC_000014.9_14261 | 0.003 | 2.77E-02 | Down |
| NC_000015.10_15591 | 0.018 | 1.77E-02 | Down |
| NC_000016.10_15636 | 0.208 | 4.53E-02 | Down |
| NC_000017.11_16813 | 0.000 | 3.04E-03 | Down |
| NC_000020.11_18728 | Inf | 2.98E-02 | Up |

Table S3. dPEMs in GC

| **Clinical pathological feature** | **dPEM** | **Expression in HC** | **Expression in GC** | **Dysregulation** |
| --- | --- | --- | --- | --- |
| Pathological TNM stage | hsa-miR-3661 | 0.45±0.48 | 0.00±0.00 | down-regulated in Stage I+II and Stage III+IV, lower than HC |
|  | hsa-miR-6716-3p | 0.51±0.77 | 0.00±0.00 | down-regulated in Stage I+II and Stage III+IV, lower than HC |
|  | hsa-miR-196a-5p | 0.02±0.03 | 0.49±0.95 | up-regulated in Stage I+II and Stage III+IV, higher than HC and Stage I+II |
|  | hsa-miR-200a-3p | 2.17±2.30 | 36.66±103.69 | up-regulated in Stage I+II and Stage III+IV, higher than HC and Stage I+II |
|  | hsa-miR-4670-5p | 0.27±0.26 | 0.20±0.59 | down-regulated in Stage I+II, lower than HC and Stage III+IV |
| Primary tumor invasion depth | hsa-miR-500a-5p | 1.22±1.46 | 0.27±0.76 | down-regulated in T_1-2_, lower than HC and T_3-4_ |
|  | hsa-miR-6861-5p | 0.00±0.00 | 0.49±1.65 | up-regulated in T_1-2_, higher than HC and T_3-4_ |
|  | hsa-miR-27a-5p | 0.00±0.00 | 0.54±0.83 | up-regulated in T_3-4_, higher than HC and T_1-2_ |
| Lymph node metastasis | hsa-miR-451a | 22412.09±11496.33 | 12185.53±11983.05 | down-regulated in N_1-3_, lower than HC and N_0_ |
|  | hsa-miR-6858-5p | 0.57±0.99 | 0.56±2.09 | down-regulated in N_1-3_, lower than HC and N_0_ |
|  | hsa-miR-302b-3p | 1.12±1.52 | 1.54±4.68 | down-regulated in N_0_, lower than HC and N_1-3_ |
|  | hsa-miR-6861-5p | 0.00±0.00 | 0.49±1.65 | up-regulated in N_0_, higher than HC and N_1-3_ |
| Distant metastasis | hsa-miR-1224-5p | 0.00±0.00 | 0.98±3.57 | up-regulated in M_1_, higher than HC and M_0_ |
|  | hsa-miR-200a-5p | 0.00±0.00 | 0.63±2.65 | up-regulated in M_1_, higher than HC and M_0_ |
|  | hsa-miR-3131 | 0.00±0.00 | 0.90±2.99 | up-regulated in M_1_, higher than HC and M_0_ |
|  | hsa-miR-320d | 2.66±2.71 | 11.61±26.78 | up-regulated in M_1_, higher than HC and M_0_ |
|  | hsa-miR-3613-5p | 5.87±5.53 | 9.16±19.42 | up-regulated in M_1_, higher than HC and M_0_ |
|  | hsa-miR-371a-3p | 0.02±0.03 | 1.06±4.17 | up-regulated in M_1_, higher than HC and M_0_ |
|  | hsa-miR-371a-5p | 0.10±0.14 | 24.44±117.24 | up-regulated in M_1_, higher than HC and M_0_ |
|  | hsa-miR-372-3p | 0.00±0.00 | 2.71±12.74 | up-regulated in M_1_, higher than HC and M_0_ |

Table S4. Comparison between dPEMs and miRNAs from GC tissues

| **dPEM** | **Exosome in plasma** | |  | **Tumor Tissue (TCGA dataset)** | |
| --- | --- | --- | --- | --- | --- |
|  | **comparison** | **dysregulation** |  | **comparison** | **dysregulation** |
| hsa-miR-451a | HC / N_0_ vs N_1-3_ | Down |  | ADJ vs Tumor | Down |
| hsa-miR-372-3p | HC / M_0_ vs M_1_ | Up |  | ADJ vs Tumor | Up |
| hsa-miR-371a-5p | HC / M_0_ vs M_1_ | Up |  | ADJ vs Tumor | Up |
| hsa-miR-371a-3p | HC / M_0_ vs M_1_ | Up |  | ADJ vs Tumor | Up |
| hsa-miR-3131 | HC / M_0_ vs M_1_ | Up |  | ADJ vs Tumor | Up |
| hsa-miR-302b-3p | HC / M_0_ vs M_1_ | Up |  | ADJ vs Tumor | Up |
| hsa-miR-200a-5p | HC / M_0_ vs M_1_ | Up |  | ADJ vs Tumor | Up |
| hsa-miR-200a-3p | HC / Stage I+II vs Stage III+IV | Up |  | ADJ vs Tumor | Up |
| hsa-miR-196a-5p | HC / Stage I+II vs Stage III+IV | Up |  | ADJ vs Tumor | Up |

Table S5. Correlation among PEMs, modules and clinical pathological features.

| **Module** | **Number of PEMs** | **Related clinical pathological features** |  | **Correlation between modules and clinical pathological features** | |  | **Correlation between MM and GS** | |
| --- | --- | --- | --- | --- | --- | --- | --- | --- |
|  |  |  |  | ***Cor*** | **P value** |  | ***Cor*** | **P value** |
| BLACK | 69 | - |  | - | - |  | - | - |
| BLUE | 164 | Distant metastasis |  | 0.45 | 2.00E-02 |  | 0.6 | 2.10E-17 |
| BROWN | 91 | - |  | - | - |  | - | - |
| GREEN | 78 | - |  | - | - |  | - | - |
| GREENYELLOW | 42 | Primary tumor invasion depth |  | -0.40 | 3.00E-02 |  | 0.41 | 7.00E-03 |
| MAGENTA | 67 | Primary tumor invasion depth |  | -0.39 | 3.00E-02 |  | -0.024 | 8.50E-01 |
| PINK | 135 | - |  | - | - |  | - | - |
| RED | 72 | - |  | - | - |  | - | - |
| TURQUOISE | 187 | - |  | - | - |  | - | - |
| YELLOW | 85 | Distant metastasis |  | 0.42 | 2.00E-02 |  | 0.64 | 4.30E-11 |
| GRAY | 142 | - |  | - | - |  | - | - |

Table S6. Hub miRNAs in BLUE and YELLOW

| **Module** | **Hub miRNAs** | **Connectivity** |  | **Correlation with distant metastasis** | |  | **Module Membership** | |
| --- | --- | --- | --- | --- | --- | --- | --- | --- |
|  |  |  |  | **GS** | **P value** |  | **MM** | **P value** |
| BLUE | hsa-miR-3613-5p | 28.06 |  | 0.52 | 3.81E-03 |  | 0.95 | 1.67E-15 |
|  | hsa-miR-148a-3p | 31.23 |  | 0.52 | 3.82E-03 |  | 0.97 | 2.09E-18 |
|  | hsa-miR-335-5p | 32.89 |  | 0.50 | 6.10E-03 |  | 0.97 | 2.69E-18 |
|  | hsa-miR-556-5p | 30.48 |  | 0.47 | 9.89E-03 |  | 0.96 | 3.80E-16 |
|  | hsa-miR-15b-5p | 28.24 |  | 0.46 | 1.23E-02 |  | 0.95 | 1.24E-15 |
|  | hsa-miR-136-3p | 29.50 |  | 0.42 | 2.34E-02 |  | 0.95 | 2.62E-15 |
|  | hsa-miR-340-5p | 31.75 |  | 0.40 | 2.99E-02 |  | 0.96 | 1.65E-16 |
|  | hsa-miR-665 | 30.56 |  | 0.40 | 3.35E-02 |  | 0.95 | 2.71E-15 |
|  | hsa-miR-130a-3p | 31.43 |  | 0.39 | 3.57E-02 |  | 0.97 | 3.08E-17 |
| YELLOW | hsa-miR-192-5p | 25.06 |  | 0.43 | 2.10E-02 |  | 0.98 | 3.83E-21 |
|  | hsa-miR-194-5p | 25.59 |  | 0.43 | 2.12E-02 |  | 0.99 | 9.16E-23 |
|  | NC_000008.11_9514 | 25.18 |  | 0.41 | 2.55E-02 |  | 0.98 | 1.51E-21 |
|  | hsa-miR-375 | 24.91 |  | 0.40 | 3.04E-02 |  | 0.99 | 8.41E-24 |
|  | hsa-miR-200c-3p | 24.19 |  | 0.39 | 3.68E-02 |  | 0.99 | 2.29E-22 |

Table S7. PEM co-expressed with hsa-miR-3613-5p in BLUE module

| **PEM** | ***ω_ij_*** |  | **Module Membership** | |  | **Correlation with distant metastasis** | |  |
| --- | --- | --- | --- | --- | --- | --- | --- | --- |
|  |  |  | **MM** | **P value** |  | **GS** | **P value** |  |
| hsa-miR-1185-1-3p | 0.39 |  | 0.91 | 5.42E-12 |  | 0.45 | 1.54E-02 |  |
| hsa-miR-1185-2-3p | 0.40 |  | 0.86 | 3.01E-09 |  | 0.32 | 9.00E-02 |  |
| hsa-miR-1283 | 0.41 |  | 0.94 | 2.54E-14 |  | 0.38 | 4.09E-02 |  |
| hsa-miR-130a-3p | 0.38 |  | 0.97 | 3.08E-17 |  | 0.39 | 3.57E-02 |  |
| hsa-miR-130b-3p | 0.36 |  | 0.90 | 2.27E-11 |  | 0.48 | 7.74E-03 |  |
| hsa-miR-136-3p | 0.30 |  | 0.95 | 2.62E-15 |  | 0.42 | 2.34E-02 |  |
| hsa-miR-148a-3p | 0.32 |  | 0.97 | 2.09E-18 |  | 0.52 | 3.82E-03 |  |
| hsa-miR-15a-5p | 0.31 |  | 0.80 | 1.41E-07 |  | 0.31 | 1.02E-01 |  |
| hsa-miR-15b-5p | 0.33 |  | 0.95 | 1.24E-15 |  | 0.46 | 1.23E-02 |  |
| hsa-miR-16-5p | 0.32 |  | 0.94 | 6.53E-14 |  | 0.44 | 1.74E-02 |  |
| hsa-miR-24-3p | 0.36 |  | 0.87 | 9.05E-10 |  | 0.36 | 5.77E-02 |  |
| hsa-miR-28-5p | 0.37 |  | 0.92 | 8.26E-13 |  | 0.44 | 1.83E-02 |  |
| hsa-miR-296-3p | 0.41 |  | 0.93 | 2.20E-13 |  | 0.39 | 3.77E-02 |  |
| hsa-miR-3140-5p | 0.34 |  | 0.90 | 1.89E-11 |  | 0.40 | 3.23E-02 |  |
| hsa-miR-3180-3p | 0.42 |  | 0.94 | 6.12E-14 |  | 0.41 | 2.56E-02 |  |
| hsa-miR-320b | 0.39 |  | 0.86 | 1.99E-09 |  | 0.39 | 3.48E-02 |  |
| hsa-miR-335-5p | 0.33 |  | 0.97 | 2.69E-18 |  | 0.50 | 6.10E-03 |  |
| hsa-miR-340-5p | 0.31 |  | 0.96 | 1.65E-16 |  | 0.40 | 2.99E-02 |  |
| hsa-miR-371a-3p | 0.41 |  | 0.93 | 1.59E-13 |  | 0.44 | 1.71E-02 |  |
| hsa-miR-371a-5p | 0.43 |  | 0.94 | 5.71E-14 |  | 0.41 | 2.58E-02 |  |
| hsa-miR-371b-3p | 0.43 |  | 0.94 | 6.05E-14 |  | 0.41 | 2.62E-02 |  |
| hsa-miR-372-3p | 0.43 |  | 0.94 | 7.22E-14 |  | 0.41 | 2.58E-02 |  |
| hsa-miR-373-3p | 0.43 |  | 0.94 | 5.85E-14 |  | 0.41 | 2.61E-02 |  |
| hsa-miR-379-3p | 0.39 |  | 0.94 | 1.04E-13 |  | 0.36 | 5.17E-02 |  |
| hsa-miR-380-3p | 0.35 |  | 0.92 | 1.21E-12 |  | 0.59 | 7.34E-04 |  |
| hsa-miR-381-3p | 0.36 |  | 0.94 | 3.28E-14 |  | 0.50 | 6.06E-03 |  |
| hsa-miR-494-3p | 0.42 |  | 0.94 | 4.49E-14 |  | 0.42 | 2.47E-02 |  |
| hsa-miR-431-3p | 0.35 |  | 0.70 | 2.53E-05 |  | 0.37 | 4.96E-02 |  |
| hsa-miR-499a-5p | 0.33 |  | 0.83 | 2.29E-08 |  | 0.36 | 5.36E-02 |  |
| hsa-miR-539-3p | 0.36 |  | 0.91 | 7.37E-12 |  | 0.49 | 7.18E-03 |  |
| hsa-miR-556-5p | 0.32 |  | 0.96 | 3.80E-16 |  | 0.47 | 9.89E-03 |  |
| hsa-miR-665 | 0.43 |  | 0.95 | 2.71E-15 |  | 0.40 | 3.35E-02 |  |
| hsa-miR-671-5p | 0.44 |  | 0.94 | 1.46E-14 |  | 0.40 | 2.95E-02 |  |
| NT_187562.1_21376_star | 0.34 |  | 0.92 | 3.30E-12 |  | 0.40 | 3.15E-02 |  |
| NT_187576.1_21403 | 0.31 |  | 0.86 | 1.84E-09 |  | 0.34 | 7.03E-02 |  |
| NT_187633.1_21617 | 0.36 |  | 0.92 | 3.48E-12 |  | 0.34 | 6.86E-02 |  |
| NT_187680.1_21728 | 0.36 |  | 0.85 | 3.59E-09 |  | 0.33 | 7.74E-02 |  |
| NW_003315906.1_21302 | 0.40 |  | 0.92 | 3.66E-12 |  | 0.42 | 2.29E-02 |  |


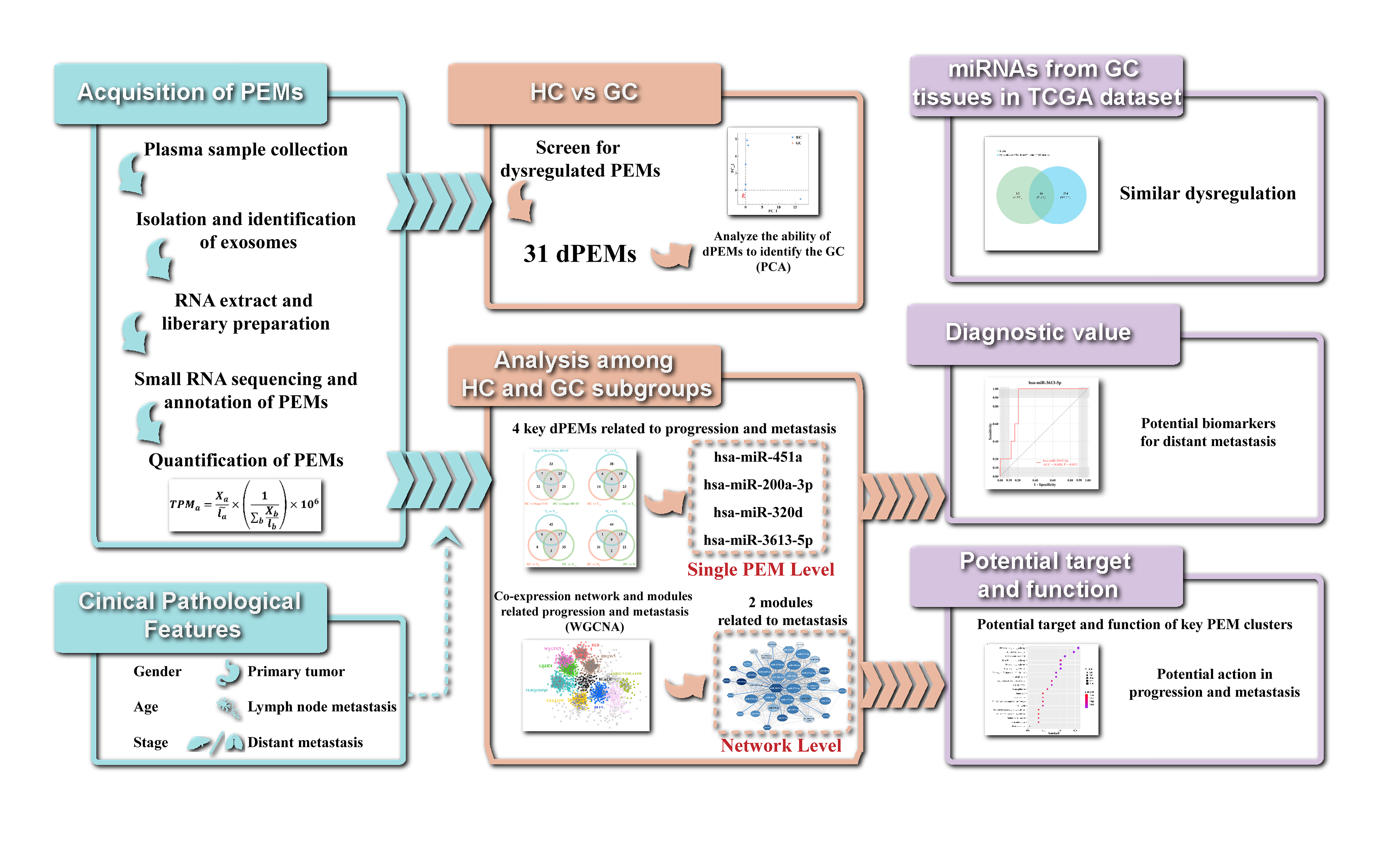


Figure S 1. The workflow of our work.


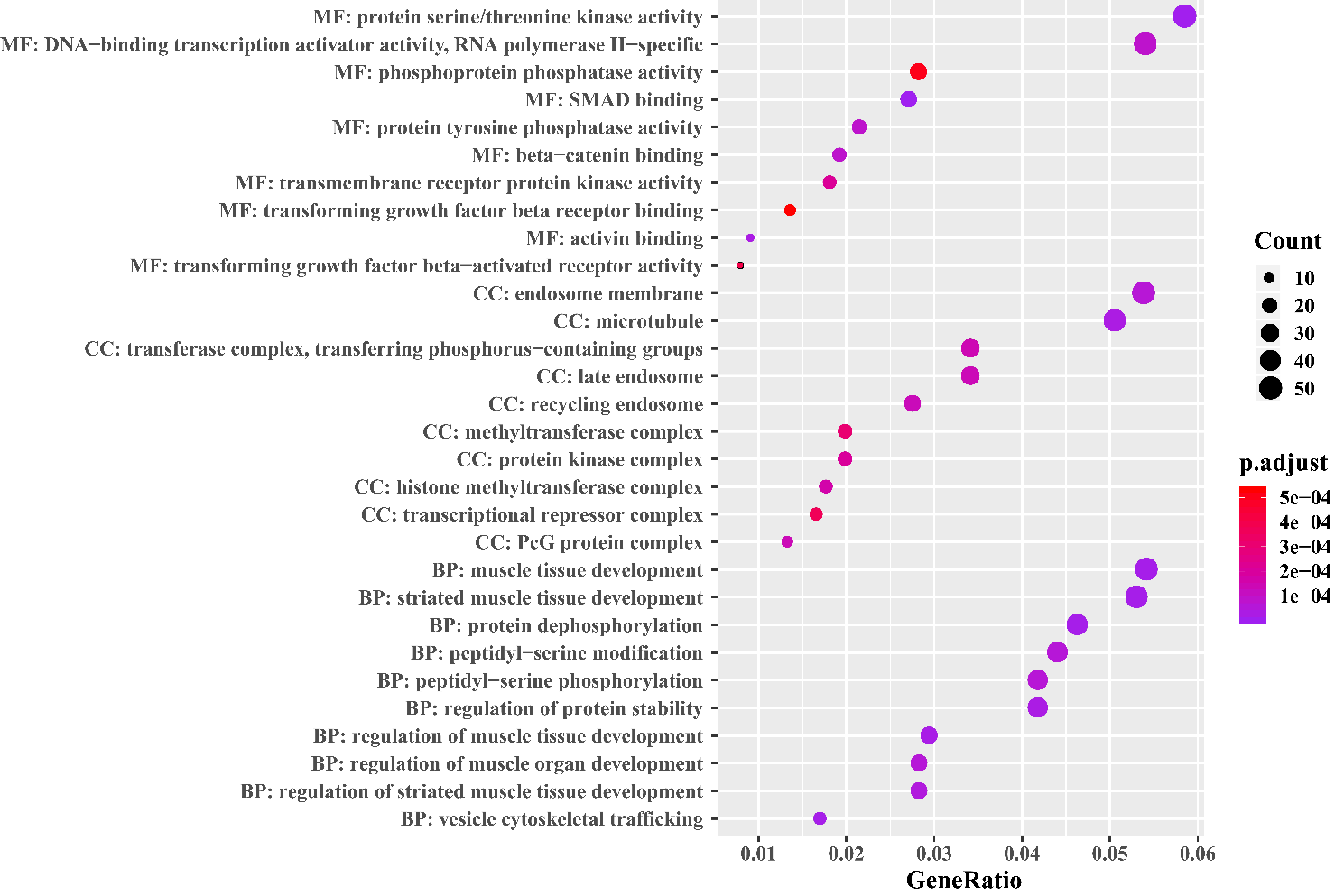


Figure S2. GO annotation of target genes of exosomal hsa-miR-3613-5p and its co-expressed PEMs. The figure above showed the Top 10 enriched GO terms in BP, CC and MF, respectively.


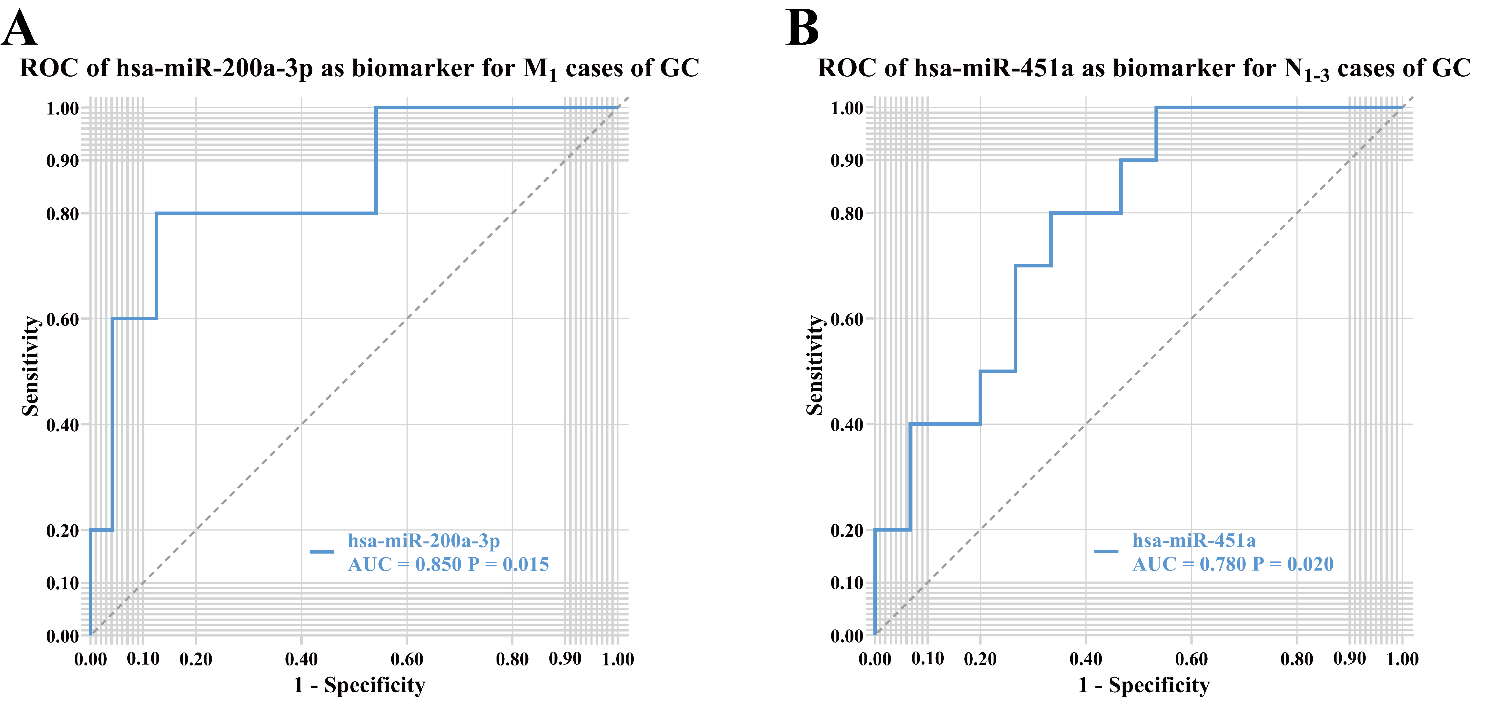


Figure S3. A, ROC of dPEM hsa-miR-200a-3p for diagnosis of GC with distant metastasis. ROC–AUC of miR-200a-3p was 0.850 (0.655-1.000, p-value = 0.015). The optimal sensitivity and specificity of miR-200a-3p was 80.0% and 87.5%, respectively, with a cut-off value of 5.444. B, ROC of dPEM hsa-miR-451a for diagnosis of GC with lymph node metastasis. ROC–AUC of miR-451a was 0.780 (0.600-0.960, p-value = 0.020). The optimal sensitivity and specificity of hsa-miR-451a was 84.4% and 66.7%, respectively, with a cut-off value of 11287.5.
