# Supplementary figures and images for: Evaluation of plasma exosomal microRNAs as circulating biomarkers for progression and metastasis of gastric cancer
Source: Clin Transl Med. 2020 Oct 11;10(6):e171. doi: 10.1002/ctm2.171 (PMC7548098; doi:10.1002/ctm2.171)

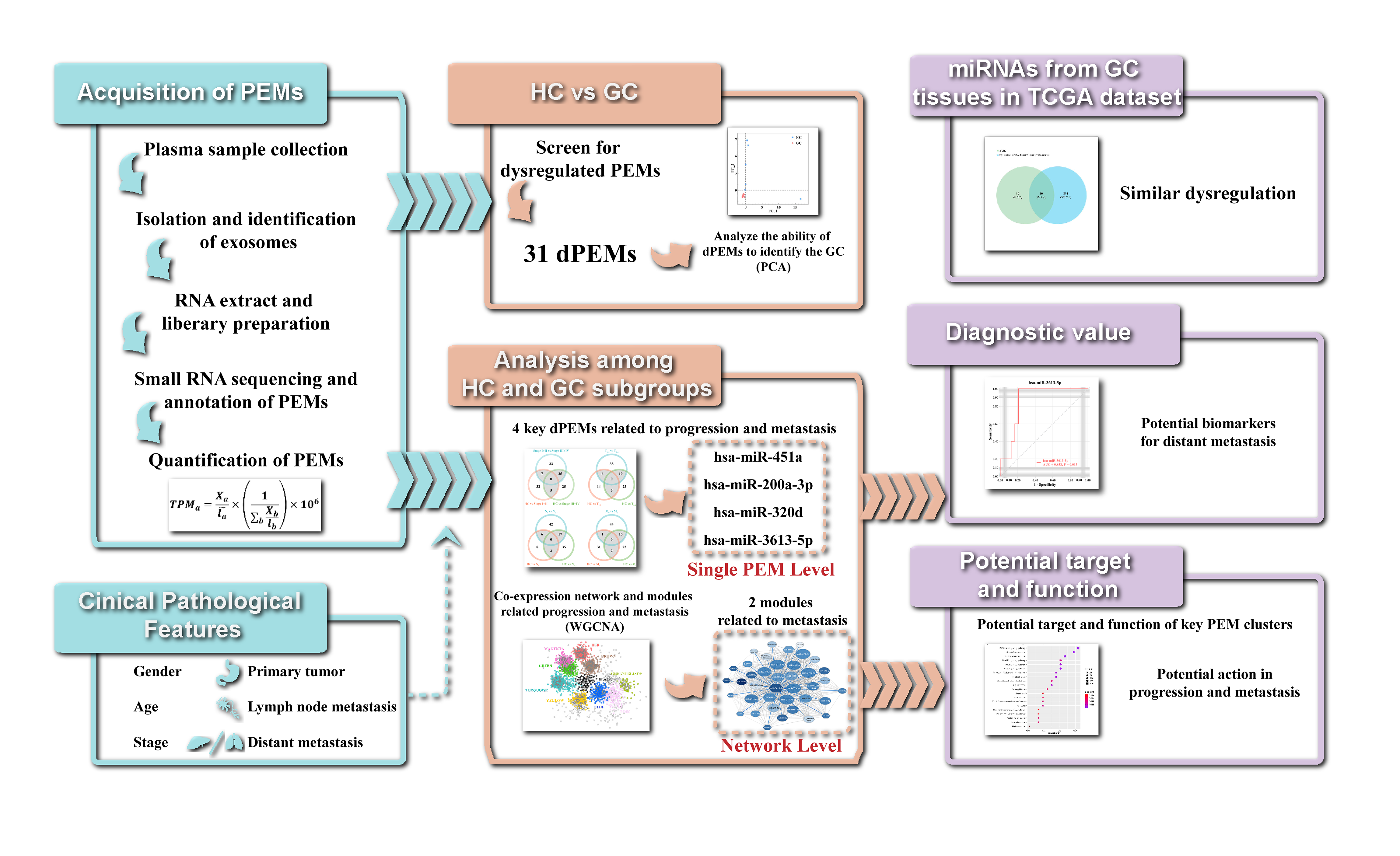

Supplement: Supplementary file 2 — Figure 1 [file CTM2-10-e171-s002.png]

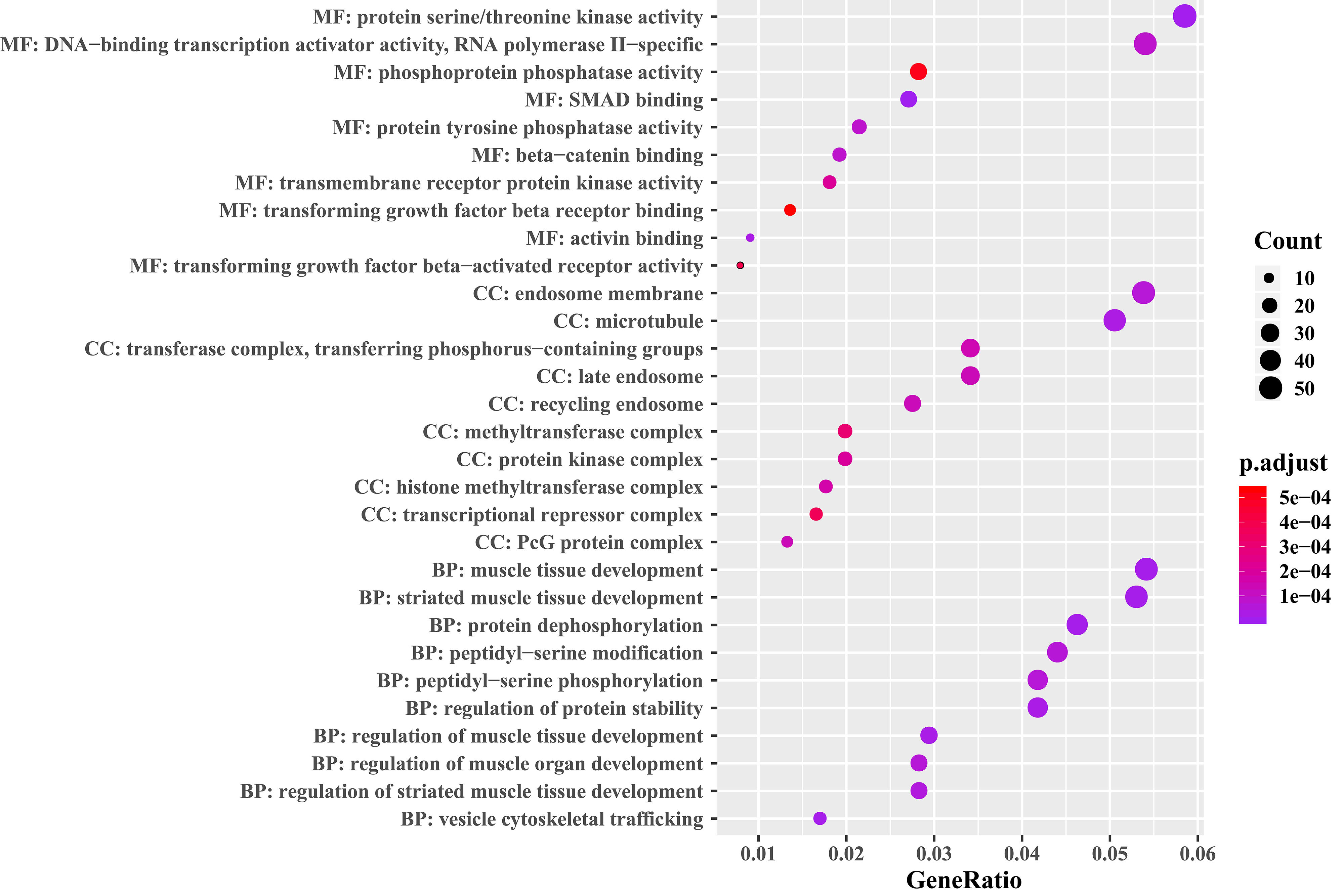

Supplement: Supplementary file 3 — Figure 2 [file CTM2-10-e171-s003.png]

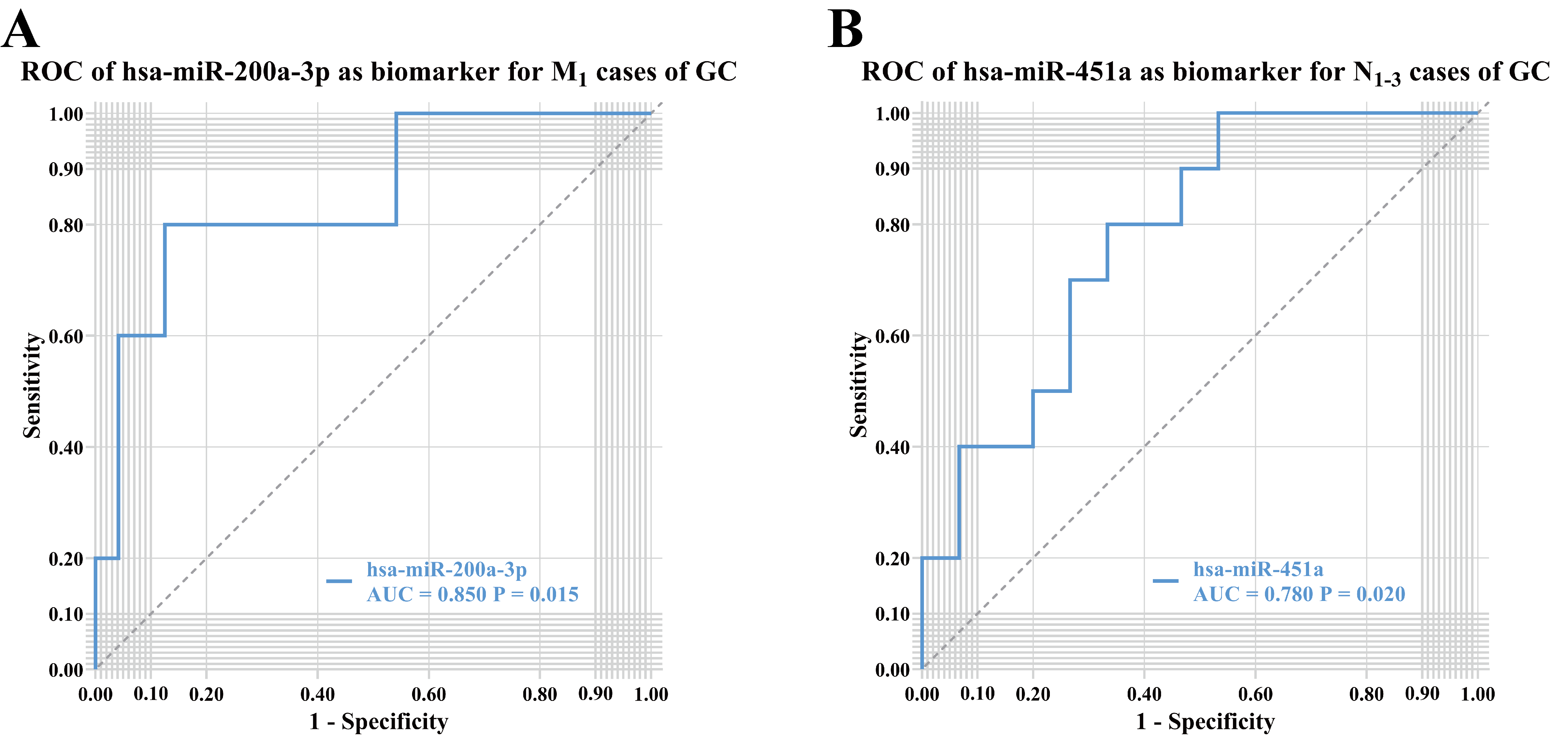

Supplement: Supplementary file 4 — Figure 3 [file CTM2-10-e171-s004.png]
